# ZnPCl_7_: a compositionally and structurally unprecedented metal–phospho­rus halide

**DOI:** 10.1107/S2056989026000174

**Published:** 2026-01-20

**Authors:** Hyeonjin Seo, Seung-Tae Hong

**Affiliations:** a Daegu Gyeongbuk Institute of Science and Technology (DGIST), Daegu 42988, Republic of Korea; b Department of Chemistry and Chemical Biology, University of New Mexico Albuquerque New Mexico 87131 USA; University of Aberdeen, United Kingdom

**Keywords:** crystal structure, zinc phospho­rus chloride, zinc(II) chloride, phospho­rus(V) chloride, moisture sensitive

## Abstract

The synthesis and crystal structure of an unprecedented compound, zinc phospho­rus hepta­chloride or *catena*-poly[phospho­rus tetra­chloride [[di­chlor­ido­zinc]-μ-chlorido]], ZnPCl_7_ or {[PCl_4_][ZnCl_3_]}_*n*_, are reported. The reaction of ZnCl_2_ with PCl_5_ in a 1:1 molar ratio at 623 K produced single crystals of ZnPCl_7_, which crystallizes in the ortho­rhom­bic space group *Ama2*. The Zn and P atoms lie on crystallographic mirror planes, as do four of the Cl atoms. One Cl atom lies on a twofold axis and one occupies a general position. Its extended structure features isolated [PCl_4_]^+^ tetra­hedra and one-dimensional chains of corner-sharing ZnCl_2_Cl_2/2_ tetra­hedra. Bond-valence sum calculations support the assignment of formal oxidation states. This discovery extends the compositional and structural landscape of mixed-metal halides.

## Abstract 2

The reaction of ZnCl_2_ with PCl_5_ in a 1:1 molar ratio at 623 K produced single crystals of ZnPCl_7_, which crystallizes in the ortho­rhom­bic space group *Ama2*. Its extended structure features isolated [PCl_4_]^+^ tetra­hedra and one-dimensional chains of corner-sharing ZnCl_2_Cl_2/2_ tetra­hedra.

## 1. Chemical context

Metal–phospho­rus compounds have garnered inter­est owing to their diverse structures and potential functional properties (Chen *et al.*, 2023 ▸). In pursuit of discovering new compounds comprising a divalent metal and phospho­rus within a halide framework, we investigated the solid-state reaction between zinc chloride (ZnCl_2_) and phospho­rus penta­chloride (PCl_5_). Heating a 1:1 stoichiometric mixture yielded a previously unreported compound, ZnPCl_7_ (**I**), confirmed by a new powder X-ray diffraction pattern. Single-crystal growth enabled full structure determination, revealing both a new stoichiometry and a previously unobserved structure in the Zn–P–Cl chemical system.

## 2. Structural commentary

**Figure 1:**
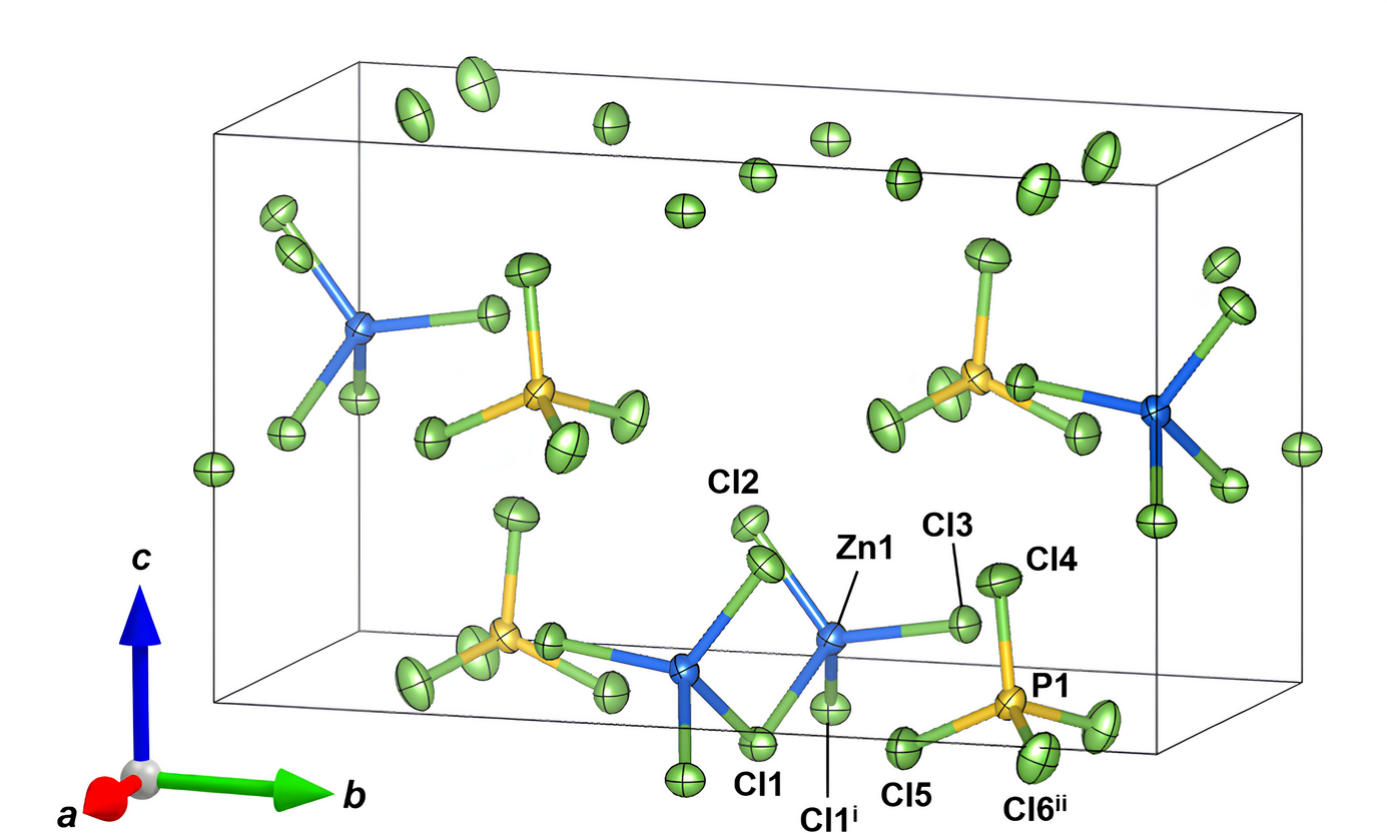
The crystal structure of (**I**) viewed approximately along the [100] direction. Displacement ellipsoids are drawn at the 50% probability level. Symmetry codes: (i) −*x* + 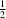, *y*, *z*; (ii) −*x* + 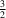, *y*, *z*.

Compound (**I**) crystallizes in the ortho­rhom­bic space group *Ama2*, with one Zn (site symmetry *m*), one P (site symmetry *m*) and six Cl atoms (four with site symmetry *m*, one with site symmetry *2* and one on a general position) in the asymmetric unit (Fig. 1 ▸). The structure can therefore be formulated as ZnPCl_7_ or [ZnCl_3_]^−^_*n*_.*n*[PCl_4_]^+^.

The Zn^2+^ ion is tetra­hedrally coordinated by four chloride anions (Table 1 ▸), with an average Zn—Cl bond distance of 2.2900 Å and Cl—Zn—Cl bond angles in the range 98.49 (4) to 118.60 (6)°. The P^5+^ atom forms a discrete [PCl_4_]^+^ tetra­hedron with a shorter average P—Cl bond distance of 1.9286 Å and Cl—P—Cl angles clustered between 109.51 (9) and 110.20 (6)°. These bond lengths are consistent with expectations based on the sums of ionic radii (Shannon, 1976 ▸).

**Figure 2:**
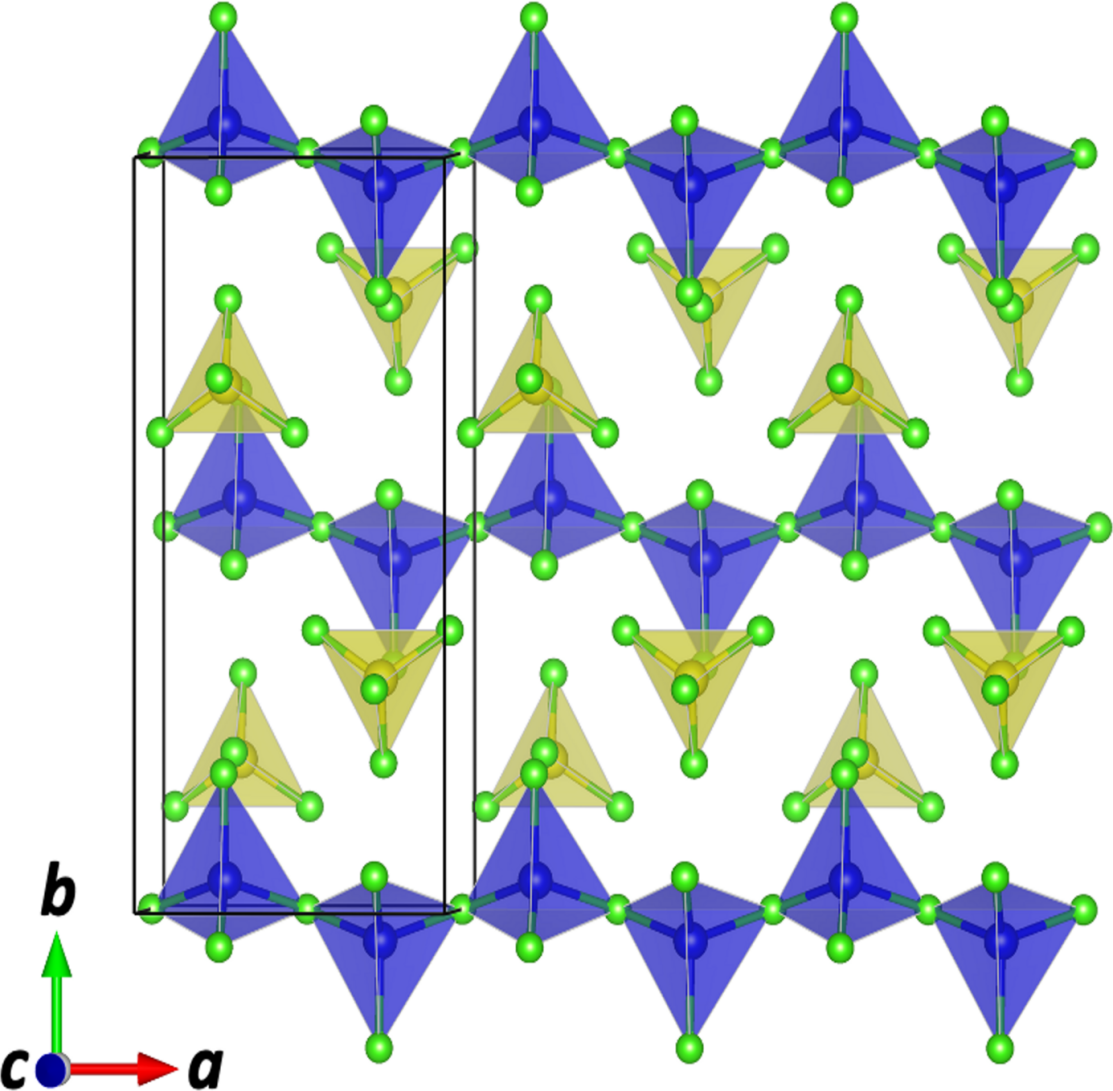
One-dimensional chains of corner-sharing ZnCl_2_Cl_2/2_ tetra­hedra (blue) extending along the *a*-axis direction in the crystal structure of (**I**). The isolated PCl_4_ tetra­hedra are depicted in yellow.

The extended structure of (**I**) consists of isolated PCl_4_ units inter­spersed between infinite [100] chains of corner-sharing ZnCl_2_Cl_2/2_ tetra­hedra (Fig. 2 ▸), forming a structure that is, to our knowledge, unprecedented in metal–halide chemistry. Such a configuration – linking *M*^2+^ centered tetra­hedra into chains and combining them with isolated *X*^5+^-centered tetra­hedra – has not been previously reported. To further validate the structural model, bond-valence-sum (BVS) calculations were performed using the *softBV* program (Chen *et al.*, 2019 ▸). The BVS values are in good agreement with the expected formal charges, further supporting the reliability of the refined structure: Zn +1.99, P +4.90, Cl1 −0.84, Cl2 −0.64, Cl3 −0.67, Cl4 −1.18, Cl5 −1.20 and Cl6 −1.17.

This type of one-dimensional chain of corner-sharing ZnCl_4_ tetra­hedra found in (**I**) is rarely observed in the halide system. Most known zinc chlorides, including ZnCl_2_, form extended networks or layered structures rather than chains (Winkler *et al.*, 1959 ▸). Similar tetra­hedral chain motifs have been reported in some oxides such as Sr_2_Fe_2_O_5_ (D’Hondt *et al.*, 2008 ▸), but are extremely uncommon among halides.

## 3. Synthesis and crystallization

Anhydrous zinc chloride (Sigma-Aldrich, 98%) and phospho­rus(V) chloride (Sigma-Aldrich, 95%) were used as received. A 1:1 molar mixture of ZnCl_2_ (0.3952 g) and PCl_5_ (0.6039 g) was thoroughly ground in an agate mortar and pressed into a pellet. The pellet was placed in a dried fused-silica ampoule, sealed under vacuum (∼360 Pa), and heated from 303 K to 623 K at a rate of 5 K min^−1^, and then slowly cooled to 373 K at a rate of 0.42 K min^−1^, followed by natural cooling to room temperature.

Single crystals were isolated under an optical microscope in a dry room with a dew point of 223 K. ZnPCl_7_ appears to be stable under dry-air conditions; therefore, all handling was carried out in a dry room. However, it is extremely sensitive to moisture and decomposed immediately upon exposure to humidity. A colourless crystal, approximately 0.1 mm in size, was placed into a 0.5 mm diameter glass capillary and sealed with capillary wax.

## 4. Refinement

Crystal data, data collection, and refinement parameters are summarized in Table 2 ▸.

## Supplementary Material

Crystal structure: contains datablock(s) global, New_Global_Publ_Block, I. DOI: 10.1107/S2056989026000174/hb8172sup1.cif

Structure factors: contains datablock(s) I. DOI: 10.1107/S2056989026000174/hb8172Isup2.hkl

CCDC reference: 2521177

Additional supporting information:  crystallographic information; 3D view; checkCIF report

## Figures and Tables

**Table 1:** Selected geometric parameters (Å, °)

Zn1—Cl1	2.3689 (8)	P1—Cl4	1.937 (2)
Zn1—Cl2	2.2066 (14)	P1—Cl5	1.924 (2)
Zn1—Cl3	2.2223 (14)	P1—Cl6	1.9250 (13)
			
Cl1^i^—Zn1—Cl1	98.49 (4)	Cl6^ii^—P1—Cl6	107.69 (9)
Cl1—Zn1—Cl2	108.41 (3)	Cl4—P1—Cl6	110.20 (6)
Cl1—Zn1—Cl3	110.52 (3)	Cl5—P1—Cl6	109.61 (6)
Cl2—Zn1—Cl3	118.60 (6)	Zn1—Cl1—Zn1^iii^	106.48 (5)
Cl4—P1—Cl5	109.51 (9)		

Symmetry codes: (i) 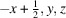; (ii) 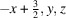; (iii) 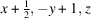.

**Table 2:** Experimental details

Crystal data	
Chemical formula	[PCl_4_][ZnCl_3_]
*M*_r_	344.51
Crystal system, space group	Orthorhombic, *Ama*2
Temperature (K)	293
*a*, *b*, *c* (Å)	7.1775 (5), 15.5212 (8), 9.0050 (4)
*V* (Å^3^)	1003.19 (10)
*Z*	4
Radiation type	Mo *K*α
μ (mm^−1^)	4.39
Crystal size (mm)	0.10 × 0.10 × 0.10

Data collection	
Diffractometer	Bruker D8 VENTURE
Absorption correction	Multi-scan (*DENZO*/*SCALEPACK*; Otwinowski & Minor, 1997 ▸)
*T*_min_, *T*_max_	0.64, 0.64
No. of measured, independent and observed [*I* > 2.0σ(*I*)] reflections	19802, 1627, 1467
*R*_int_	0.050
(sin θ/λ)_max_ (Å^−1^)	0.712

Refinement	
*R*[*F*^2^ > 2σ(*F*^2^)], *wR*(*F*^2^), *S*	0.028, 0.060, 1.29
No. of reflections	1627
No. of parameters	52
No. of restraints	17
Δρ_max_, Δρ_min_ (e Å^−3^)	0.58, −0.55
Absolute structure	Parsons et al. (2013 ▸), 764 Friedel Pairs
Absolute structure parameter	0.016 (17)

Computer programs: *APEX2* and *SAINT* (Bruker, 2006 ▸), *SUPERFLIP* (Palatinus & Chapuis, 2007 ▸), *CRYSTALS* (Betteridge *et al.*, 2003 ▸) and *VESTA* (Momma & Izumi, 2011 ▸).

## supplementary crystallographic information

### (I) Crystal data

**Table:**

[PCl_4_][ZnCl_3_]	*F*(000) = 656
*M**_r_* = 344.51	*D*_x_ = 2.281 Mg m^−^^3^
Orthorhombic, *A**m**a*2	Mo *K*α radiation, λ = 0.71073 Å
Hall symbol: A 2 -2a	Cell parameters from 1767 reflections
*a* = 7.1775 (5) Å	θ = 3.9–31.3°
*b* = 15.5212 (8) Å	µ = 4.39 mm^−^^1^
*c* = 9.0050 (4) Å	*T* = 293 K
*V* = 1003.19 (10) Å^3^	Block, colourless
*Z* = 4	0.10 × 0.10 × 0.10 mm

### (I) Data collection

**Table:**

Bruker D8 VENTURE diffractometer	1467 reflections with *I* > 2.0σ(*I*)
Graphite monochromator	*R*_int_ = 0.050
ω/2θ scans	θ_max_ = 30.4°, θ_min_ = 3.9°
Absorption correction: multi-scan (DENZO/SCALEPACK; Otwinowski & Minor, 1997)	*h* = −10→10
*T*_min_ = 0.64, *T*_max_ = 0.64	*k* = −21→22
19802 measured reflections	*l* = −12→12
1627 independent reflections	

### (I) Refinement

**Table:**

Refinement on *F*^2^	Primary atom site location: other
Least-squares matrix: full	Method, part 1, Chebychev polynomial, (Watkin, 1994, *P*rince, 1982) [*w*eight] = 1.0/[A_0_*T_0_(x) + A_1_*T_1_(x) ··· + A_n-1_]*T_n-1_(x)] where A_i_ are the Chebychev coefficients listed belo*w* and x = *F* /*F*max Method = Robust Weighting (*P*rince, 1982) W = [*w*eight] * [1-(delta*F*/6*sigma*F*)^2^]^2^ A_i_ are: 20.3 28.2 14.3 4.23 0.510
*R*[*F*^2^ > 2σ(*F*^2^)] = 0.028	(Δ/σ)_max_ = 0.0003
*wR*(*F*^2^) = 0.060	Δρ_max_ = 0.58 e Å^−^^3^
*S* = 1.29	Δρ_min_ = −0.55 e Å^−^^3^
1627 reflections	Absolute structure: Parsons et al. (2013), 764 Friedel Pairs
52 parameters	Absolute structure parameter: 0.016 (17)
17 restraints	

### (I) Fractional atomic coordinates and isotropic or equivalent isotropic displacement parameters (Å^2^)

**Table:**

	*x*	*y*	*z*	*U*_iso_*/*U*_eq_	
Zn1	0.2500	0.53981 (3)	0.06695 (9)	0.0283	
P1	0.7500	0.80615 (8)	0.04103 (15)	0.0301	
Cl1	0.5000	0.5000	−0.09048 (14)	0.0318	
Cl2	0.2500	0.45321 (9)	0.26129 (16)	0.0370	
Cl3	0.2500	0.68125 (8)	0.10522 (15)	0.0394	
Cl4	0.7500	0.79444 (11)	0.25514 (17)	0.0538	
Cl5	0.7500	0.69371 (10)	−0.04891 (18)	0.0503	
Cl6	0.53345 (16)	0.86924 (7)	−0.02285 (15)	0.0535	

### (I) Atomic displacement parameters (Å^2^)

**Table:**

	*U*^11^	*U*^22^	*U*^33^	*U*^12^	*U*^13^	*U*^23^
Zn1	0.0314 (3)	0.0250 (2)	0.0284 (3)	0.0000	0.0000	0.0023 (2)
P1	0.0314 (6)	0.0273 (6)	0.0316 (7)	0.0000	0.0000	0.0072 (5)
Cl1	0.0238 (5)	0.0414 (6)	0.0302 (5)	0.0025 (5)	0.0000	0.0000
Cl2	0.0380 (7)	0.0381 (7)	0.0351 (6)	0.0000	0.0000	0.0125 (6)
Cl3	0.0563 (9)	0.0257 (5)	0.0363 (7)	0.0000	0.0000	−0.0015 (5)
Cl4	0.0756 (12)	0.0546 (9)	0.0314 (7)	0.0000	0.0000	0.0058 (7)
Cl5	0.0740 (11)	0.0308 (7)	0.0461 (9)	0.0000	0.0000	0.0001 (6)
Cl6	0.0385 (5)	0.0465 (6)	0.0753 (8)	0.0087 (5)	−0.0050 (5)	0.0161 (5)

### (I) Geometric parameters (Å, º)

**Table:**

Zn1—Cl1^i^	2.3689 (8)	P1—Cl6^ii^	1.9250 (13)
Zn1—Cl1	2.3689 (8)	P1—Cl4	1.937 (2)
Zn1—Cl2	2.2066 (14)	P1—Cl5	1.924 (2)
Zn1—Cl3	2.2223 (14)	P1—Cl6	1.9250 (13)
			
Cl1^i^—Zn1—Cl1	98.49 (4)	Cl6^ii^—P1—Cl5	109.61 (6)
Cl1^i^—Zn1—Cl2	108.41 (3)	Cl4—P1—Cl5	109.51 (9)
Cl1—Zn1—Cl2	108.41 (3)	Cl6^ii^—P1—Cl6	107.69 (9)
Cl1^i^—Zn1—Cl3	110.52 (3)	Cl4—P1—Cl6	110.20 (6)
Cl1—Zn1—Cl3	110.52 (3)	Cl5—P1—Cl6	109.61 (6)
Cl2—Zn1—Cl3	118.60 (6)	Zn1—Cl1—Zn1^iii^	106.48 (5)
Cl6^ii^—P1—Cl4	110.20 (7)		

Symmetry codes: (i) −*x*+1/2, *y*, *z*; (ii) −*x*+3/2, *y*, *z*; (iii) *x*+1/2, −*y*+1, *z*.
